# *Rubus coreanus* Enhances Peri-Implant Bone Healing and Biomineralization in Ovariectomized and Healthy Rats

**DOI:** 10.3390/biology14020139

**Published:** 2025-01-29

**Authors:** Naara Gabriela Monteiro, Odir Nunes de Oliveira-Filho, Maria Isabela Lopes Gandolfo, Ana Cláudia Ervolino da Silva, Letícia Pitol-Palin, Paulo Roberto Botacin, Gabriel Mulinari-Santos, Fábio Roberto de Souza Batista, Roberta Okamoto

**Affiliations:** 1 Department of Diagnosis and Surgery, Araçatuba Dental School, São Paulo State University Júlio de Mesquita Filho, Araçatuba 16015-050, Brazil; naara.monteiro@unesp.br (N.G.M.); odir.nunes@unesp.br (O.N.d.O.-F.); isa.gandolfo@hotmail.com (M.I.L.G.); ana.ervolino@unesp.br (A.C.E.d.S.); leticia.p.palin@unesp.br (L.P.-P.); fabiorsbatista@gmail.com (F.R.d.S.B.); 2 Department of Basic Sciences, Araçatuba Dental School, São Paulo State University Júlio de Mesquita Filho, Araçatuba 16066-840, Brazil; paulo.botacin@unesp.br

**Keywords:** osteoporosis, bone, osseointegration, biomineralization, *Rubus coreanus*

## Abstract

Estrogen deficiency contributes to osteoporosis and can therefore compromise the peri-implant bone. Hence, this study evaluated peri-implant bone healing when *Rubus coreanus* (RC) was administered orally in ovariectomized and healthy rats. Forty 4-month-old female rats were divided into four groups: SHAM (healthy rats), SHAM/RC (healthy rats treated with RC), OVX (ovariectomized rats), and OVX/RC (ovariectomized rats treated with RC). The oral administration of RC started thirty days after ovariectomy, and implant placement into the rat tibia occurred ninety days after the ovariectomy. Euthanasia occurred sixty days after implantation. The analyses performed included removal torque, RT-PCR, confocal microscopy, and immunolabeling. A significance level of *p* < 0.05 was considered for all tests. The highest reverse torque values were observed in the SHAM/RC group, followed by the OVX/RC group. Confocal microscopy showed the greatest bone biomineralization in the SHAM/RC group, followed by the OVX/RC group. RT-PCR data indicated that RC decreased the RANKL/OPG ratio in both conditions. Immunohistochemistry demonstrated a balance between bone formation and resorption in all groups, especially stimulating osteoblastogenesis in both treated groups. In conclusion, RC enhanced peri-implant bone healing and biomineralization in both healthy and ovariectomized rats, with stronger effects in healthy rats, suggesting that estrogen may enhance its efficacy. These findings support RC’s potential as a prophylactic and therapeutic agent.

## Simple Summary

This study explored the impact of *Rubus coreanus* (RC) on peri-implant bone healing in rats with and without estrogen deficiency. The rats were divided into four groups: healthy (SHAM), healthy with RC treatment (SHAM/RC), ovariectomized (OVX), and ovariectomized with RC treatment (OVX/RC). RC was given 30 days after ovariectomy, with implant placement occurring 90 days later. The results showed that RC treatment improved bone healing, with the SHAM/RC group exhibiting the highest bone biomineralization and removal torque. RC also helped balance bone formation and resorption in the ovariectomized rats. These findings suggest that RC can enhance peri-implant bone healing in both healthy and ovariectomized rats, with hormonal status influencing its effectiveness. Further research is needed to investigate the mechanisms.

## 1. Introduction

Osteoporosis is a systemic skeletal disorder characterized by progressive bone loss and the deterioration of the bone microarchitecture due to an imbalance of bone formation and resorption [1,2]. These changes in the bone microarchitecture lead to a decrease in bone strength, resulting in bone fragility and an increased susceptibility to fractures [1]. Fractures most commonly occur in long bones such as the femur, radius, and lumbar spine [3]. However, patients with osteoporosis also demonstrate reduced bone mass in their skull bones [4,5]. While osteoporosis is not considered a contraindication to dental implant treatment, this condition can potentially hinder the healing and stability of the implant [6,7,8]. Therefore, effective therapeutic strategies are essential to ensure the quality of the bone surrounding the implant.

Postmenopausal bone loss can significantly impact the success of dental implants, leading to increased risks of implant failure [6,9]. In particular, postmenopausal women may require dental implants due to conditions such as periodontal disease, trauma, or alveolar bone loss leading to tooth loss [10,11]. Following dental implant placement, marginal bone loss around the implant site is a common concern [12]. Various interventions have been proposed to enhance peri-implant bone health [13,14]. Strategies such as using functionalized bone grafts and biologic agents and advancements in dental implant design and surfaces aim to enhance peri-implant bone healing and promote osseointegration [8,15]. Improving the compromised bone is crucial for achieving better outcomes in postmenopausal women undergoing dental implant treatment [8].

Treating osteoporosis typically involves antiresorptive and anabolic agents [2,16,17], which can have adverse effects, including medication-related osteonecrosis of the jaw [18]. Therefore, exploring natural alternatives to treat osteoporosis and enhance bone healing is essential. Recently, plants and their extracts have been studied for their beneficial effects and reduced adverse reactions [19,20]. *Rubus coreanus* (RC), a member of the Rosaceae family, Rosoideae subfamily, and Potentilleae tribe, is a promising alternative [21]. RC has been used for various therapeutic purposes due to its anti-inflammatory, antiviral, anti-carcinogenic, and antioxidant effects [22]. Additionally, RC has positive effects on bone, promoting the increased differentiation of osteoblasts and apoptosis of osteoclasts [20,23]. It also regulates bone remodeling by balancing bone formation and resorption [19]. Thus, RC might support peri-implant healing under osteoporotic conditions.

Estrogen deficiency is a key factor in the development of osteoporosis [24,25]. In postmenopausal women, reduced estrogen levels result in increased bone resorption, leading to decreased bone mass in individuals with osteoporosis [26,27]. In the alveolar bone, this manifests as decreased bone density [10]. Low bone density due to estrogen deficiency can contribute to implant failure [6,7,28]. The main reason is a delay in alveolar and peri-implant bone healing, which leads to a decrease in primary stability [29] and the loss of bone around implants [6]. Both factors are linked to the success of dental implant treatment [30]. Therefore, alternatives to enhance the peri-implant bone under estrogen-deficient conditions are being investigated. Thus, this study aimed to evaluate peri-implant bone healing following RC administration in healthy and ovariectomized rats. We used the rat tibia model to assess peri-implant bone healing [28,31], as it is a well-established technique for analyzing cellular responses and biomechanical properties [31,32]. These characteristics are crucial for studying bone regeneration and implant stability, making the tibial model relevant for evaluating RC’s effects on peri-implant healing and its potential dental implant implications. Additionally, ovariectomy is a recognized method for simulating estrogen deficiency [33]. This deficiency is primarily associated with alterations in bone quality, which significantly affect osseointegration [34,35,36].

## 2. Materials and Methods

### 2.1. Study Design and Ethics

The present study complies with the ARRIVE guidelines [37] and national laws on animal use. The Ethics Committee in the Use of Animals of Araçatuba Dental School (CEUA: 00410-2020) approved this study. Forty female Wistar rats (*Rattus norvegicus*, Albinus) were maintained at a temperature of 22 °C, in a 12 h light/12 h dark cycle, with balanced feed (Ração Mogiana Alimentos SA, Campinas, Brazil). The rats were selected for the experiment after confirmation of their regular estrous cycle. Rats were OVX- or SHAM-operated under general anesthesia with xylazine (0.03 mL/100 g bw/ip–Dopaser^®^ Laboratories Calier S.A., Barcelona, Spain) and ketamine (0.07mL/100 g bw/ip–Fort Dodge Saúde Animal Ltda, Campinas, São Paulo, Brazil). Animals were randomized into four groups, with 10 animals in each group, according to the treatment: SHAM (healthy rats treated with an oral gavage of saline), SHAM/RC (healthy rats treated with 200 mg/kg/day of RC by oral gavage), OVX (ovariectomized rats treated with an oral gavage of saline), and OVX/RC (ovariectomized rats treated with 200 mg/kg/day of RC by oral gavage). All treatments began 30 days after the surgery and lasted for 120 days, and this design followed that in a prior study [38].

### 2.2. RC Administration

The SHAM/RC and OVX/RC groups received RC oral administration daily of 200 mg/kg/day through oral gavage, as described in a prior study [38]. To ensure consistent drug intake, we carefully monitored the administration process, as oral gavage allows for precise dosing. Before each administration, the body weight of each rat was measured. Treatment began 30 days after ovariectomy and continued until the time of euthanasia at day 150. RC was prepared from an extract of dried *Rubus coreanus* leaves. A total of 1000 g of dried leaves was extracted at room temperature, and the extract was concentrated under reduced pressure, filtered, lyophilized, and stored at 4 °C. The resulting powdered extract was then dissolved in a 0.25% dimethyl sulfoxide solution (Aphoticario Manipulation Pharmacy, Araçatuba SP, Brazil) in normal saline for oral administration.

### 2.3. Implant Placement

Ninety days after the ovariectomy, the animals were anesthetized with xylazine hydrochloride (0.03 mL per 100 g body weight; Coopers Brasil Ltda., Cotia, SP, Brazil) and ketamine hydrochloride (0.07 mL per 100 g body weight; Fort Dodge Animal Health, Iowa, IA, USA). Moreover, local anesthesia with mepivacaine (0.3 mL/kg 2%, adrenaline 1:100,000, Septodont, Saint-Maur-des Fossés, France) was administered after antisepsis (polyvinylpyrrolidone iodide; Indústria Química e Farmacêutica Rioquímica Ltda, São José do Rio Preto, São Paulo, Brazil). Surgical access was obtained with a 1.5 cm incision in the tibial metaphysis. The soft tissue was then dissected at full thickness and removed with periosteal detachers, exposing the bone to receive the implants. Grade 4 titanium implants with a 1.5 mm diameter and 5 mm length with an acid-etched surface (Medens, Itu, São Paulo, Brazil) were installed bilaterally in each tibia with bicortical stabilization. The sutures were made with Vycril (Poliglactin 910, Ethicon, Johnson & Johnson Prod, São José dos Campos, Brazil). In the immediate postoperative period, each animal received pentabiotic (0.1 mL/kg; Fort Dodge Saúde Animal Ltda, Campinas, São Paulo, Brazil) and sodic dipyrone (1 mg/kg; Ariston, Indústrias Químicas e Farmacêuticas Ltda, São Paulo, Brazil). The implant placement followed a procedure described in a previous study [28].

### 2.4. Fluorochromes Application

**Figure 1:**
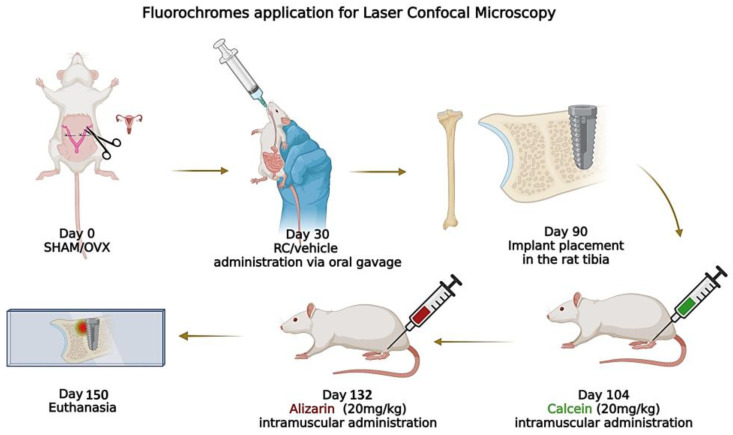
The fluorochrome application timeline for assessing bone mineralization. Animals received an initial injection of calcein (20 mg/kg) 14 days after implantation to label older bone, followed by alizarin red (20 mg/kg) 42 days post-implantation to mark new bone formation. Both fluorochromes bind to bone minerals, allowing the measurement of bone mineralization.

After 14 days of implant installation, the animals were given an intramuscular injection of calcein (20 mg/kg). Then, 42 days after the implant installation, the same animals received an intramuscular injection of alizarin red (20 mg/kg). The fluorochromes act by binding to minerals in the bone matrix. This labeling measured bone mineral precipitation. The first fluorochrome received indicates an older bone, while the second indicates a new bone, as referred to in an earlier study [28]. The timeline for the application of fluorochromes is shown in Figure 1.

### 2.5. Biomechanical Test—Removal Torque

At 60 days after implant installation, the animals were sedated with xylazine hydrochloride (0.03 mL per 100 g body weight; Coopers Brasil Ltda., Cotia, SP, Brazil) to promote muscle relaxation and ketamine hydrochloride (0.07 mL per 100 g body weight; Fort Dodge Animal Health, IA, USA) to induce anesthesia. After the right tibia was prepared with antisepsis using polyvinylpyrrolidone iodide (Indústria Química e Farmacêutica Rioquímica Ltda, Brazil), a surgical incision of approximately 1.5 cm was made in the right tibial metaphysis region. The soft tissue was then dissected at full thickness and removed with the aid of periosteal detachers, exposing the installed implant. Using a digital torquemeter, the implants were removed from the tibia by removal torque, and the values obtained were tabulated. The removal torque for each tibia was recorded by applying an increasing anticlockwise force until the implant began to rotate within the bone, with the maximum torque measured in Newton centimeters (Ncm). The removal torque procedure followed a prior method [28].

### 2.6. Molecular Analysis—RT PCR

After the removal of the implants from the tibias using removal torque, from the same tibias, the peri-implant bone of the right tibia was collected. Each fragment was washed in a PBS solution and then placed in liquid nitrogen. Total RNA was extracted using a Trizol reagent (Life Technologies Invitrogen, Carlsbad, CA, USA). Reverse transcription polymerase chain reaction (RT-PCR) was performed to assess the expression of osteoprotegerin (OPG), receptor activator of nuclear factor kappa-B ligand (RANKL), osteocalcin (OC), and alkaline phosphatase (ALP). The rat genes and the TaqMan Gene Expression Assays (Applied Biosystems, Foster City, CA, USA) of the primer/probe sets used were as follows: OPG (Tnfrsf11b, Rn00563499_m1), RANKL (Tnfrsf11, Rn00589289_ m1), OC (Bglap, Rn0056386_g1) and ALP (Alpl, Rn00564931_m1). After the integrity, purity, and concentration of the RNA was determined, cDNA was made using 1 μg of RNA in a reverse transcriptase reaction (M-MLV reverse transcriptase; Promega Corporation, Madison, WI, USA). RT-PCR was performed using a detection system for RT-PCR CFX96 (Bio-Rad Laboratories, Philadelphia, PA, USA) with the SybrGreen system (Applied Biosystems, Warrington, UK) under the following conditions: 50 °C (2 min), 95 °C (10 min) and 40 cycles of 95 °C (15 s), and 60 °C (1 min), followed by a standard denaturation curve. Relative gene expression was calculated regarding the expression of proteins in ribosomal mitochondria, and the gene expression of the peri-implant bone in the different experimental periods was normalized (ΔΔCT method). Assays were performed in quadruplicate. The ratio RANKL/OPG was calculated by dividing the RANKL levels by the OPG levels. After reverse torque and molecular analysis, the animals were euthanized by anesthesia overdose, as previously described [39]. The TaqMan probes for real-time PCR are shown in Table 1.

### 2.7. Laboratory Processing Immunohistochemical Analysis

The tibias were randomly selected for the proposed analyses. They were fixed in 10% formaldehyde for 48 h. Five tibias underwent immunohistochemical analysis after being demineralized in 10% EDTA (Merck, Darmstadt, Germany) and embedded in paraffin. The specimens were then sliced along the longitudinal axis of the implant until 5 μm slices were obtained. These slices were mounted on histological slides. The immunohistochemical analysis evaluated the proteins Runt-related transcription factor 2 (RUNX2), alkaline phosphatase (ALP), osteopontin (OPN), osteocalcin (OCN), Tartrate-resistant acid phosphatase (TRAP), osteoprotegerin (OPG), and receptor activator of nuclear factor kappa-Β ligand (RANKL). The primary goat antibodies from Santa Cruz Biotechnology (Santa Cruz, CA, USA) used included anti-Runx2 (Catalog number: SC-390351), anti-alkaline phosphatase (Catalog number: SC-365765), anti-OPN (Catalog number: SC-390518), anti-OCN (Catalog number: SC 18319), anti-TRAP (Catalog number: SC 30832), anti-OPG (Catalog number: SC 21038), and anti-RANKL (Catalog number: SC 7627). A biotinylated donkey anti-goat antibody (Jackson Immunoresearch Laboratories, West Grove, PA, USA) was used as the secondary antibody. The immunohistochemical reaction was enhanced with an avidin–biotin system (Kit ABC-Vectastain Elite ABC–peroxidase standard, reagent A and B only–PK6100; Vector Laboratories, Burlingame, CA, USA), and diaminobenzidine (Sigma, Saint Louis, MO, USA) was used as the chromogen. Immunohistochemical reactions were monitored to assess label specificity, and hematoxylin was used for counter-staining. Data analysis was conducted qualitatively, with scores ranging from 0 for the absence of labeling to 0, 1, 2, and 3 for absence, low, moderate, and intense marking, respectively. The immunohistochemistry procedures followed those in a previous study [40], where scores are attributed according to the size of positive labeling observed in the region of interest. Score 0 is attributed to the absence of positive labeling; score 1 is attributed when 25% of the region has positive labeling; score 2 is attributed when 50% of the region has positive labeling; and finally, score 3 is attributed when 75% of the region has positive labeling. The evaluation was performed by a blind calibrated researcher (R.O). It is important to highlight that the scores were attributed three times, on different days, and were considered satisfactory when a Kappa index of 80% was achieved.

### 2.8. Laboratory Processing of Laser Confocal Microscopy

This process followed that in a prior study [28]. The tibias were initially fixed in 10% formaldehyde for 48 h. Following fixation, they were dehydrated in increasing concentrations of alcohol and then soaked and infiltrated with a 1:1 solution of acetone and methyl methacrylate (MMA; Classical Dental Articles, São Paulo, Brazil), which was added slowly. The samples were then placed in three MMA baths, with 1% benzoyl peroxide catalyst (Riedel—De Haën AG, Seelze—Hannover, Germany) added to the final bath. The final polymerization was carried out by placing the samples in capped glass vials, which were maintained at 37 °C for 5 days to allow the resin to fully polymerize. After polymerization, blocks containing the specimens were initially reduced with a driller mounted on a Kota bench motor (Strong 210, São Paulo, Brazil), parallel to the long axis of the tibiae on the sagittal plane. Progressive manual wear on an automatic polisher (ECOMET 250PRO/AUTOMET 250, Buehler, Lake Bluff, IL, USA) was used. The sections were mounted on histological slides. After the sections were analyzed using a Leica CTR 4000 CS SPE microscope (Leica Microsystems, Heidelberg, Germany) under a 10× objective (original magnification of 100), the images were reconstructed, and the peri-implant bone showed the overlap of the two fluorochromes. These images were exported to ImageJ software (version 1.52v, National Institutes of Health, Bethesda, MD, USA) to measure the bone dynamics represented by the difference between old bone and new bone precipitation. Through the use of the “color selection” tool, each image was standardized according to hue, saturation, and brightness. Both bone types were observed in the same setting on a single slide for measuring the areas of alizarin and calcein fluorochrome precipitation in the medullary bone from the second to the fifth thread of the implant in μm^2^. The MAR value was calculated as the distance (µm) between the two fluorochromes per day.

### 2.9. Statistical Analysis

Statistical tests were performed using the GraphPad Prism 7 program (GraphPad Software; La Jolla, CA, USA). For the quantitative parameters from reverse torque, real-time PCR, and laser confocal microscopy, the normality and homoscedasticity tests were conducted to verify the normal distribution of the data. The Shapiro–Wilk test was used to assess normality. Based on this, a two-way ANOVA followed by Tukey tests was performed. A significance level of *p* < 0.05 was adopted.

## 3. Results

### 3.1. Biomechanical Analysis

#### RC Increases Removal Torque in Healthy and Ovariectomized Rats

**Figure 2:**
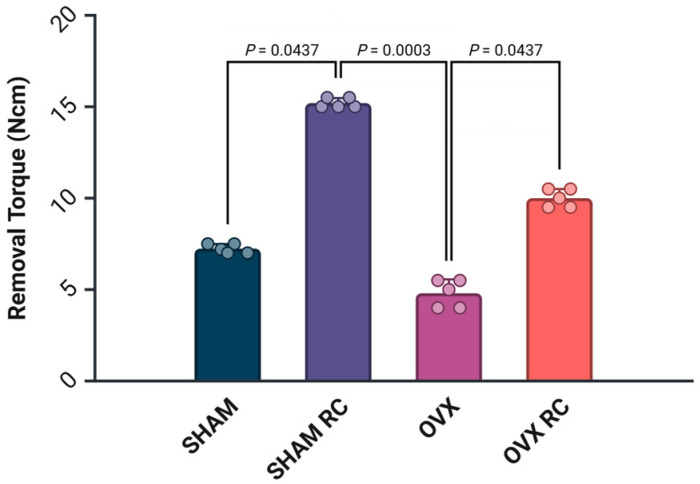
Biomechanical analysis of removal torque. The dot–bar graph shows the mean results, and the error bars indicate the standard deviation for the removal torque value from experimental groups followed by SHAM, SHAM/RC, OVX, and OVX/RC at 60 days after implantation. The *p* < 0.05 indicates statistical differences among the groups.

The median results of the biomechanical analysis were SHAM (7.6 Ncm), SHAM/RC (15.1 Ncm), OVX (4.6 Ncm), and OVX/RC (10.0 Ncm). All groups showed statistically significant differences when compared to each other (*p* < 0.05). The data indicated that the RC had a prophylactic effect on healthy rats, as shown by the values obtained in the SHAM/RC group. Additionally, the results of the OVX/RC group, which were greater than those of the OVX group, indicate a therapeutic effect (Figure 2).

### 3.2. Molecular Results—RT PCR

**Figure 3:**
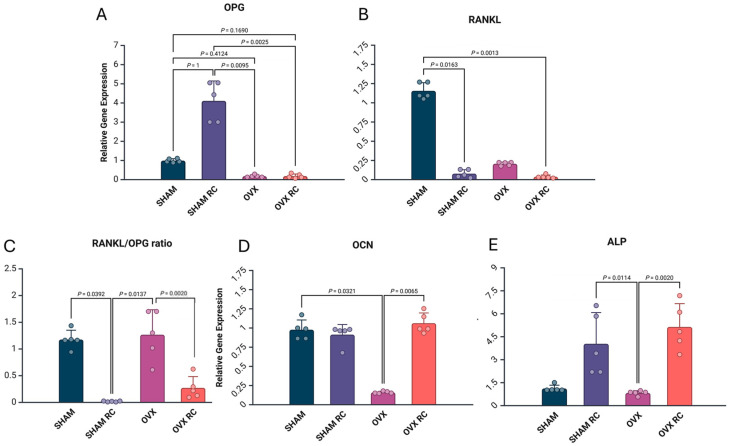
Molecular analysis for each gene expression (**A**–**E**). The dot–bar graph shows the mean results, and the error bars indicate the standard deviation for relative gene expression from the real-time PCR in peri-implant bone healing from the SHAM, SHAM/RC, OVX, and OVX/RC groups after 60 days of implantation. In RT-PCR, the genes evaluated were OPG, RANKL, OCN, and ALP. *p* < 0.05 indicates statistical differences between the groups.

Systemic treatment with RC caused an overexpression of OPG in healthy treated rats compared to untreated healthy rats (***p*** < 0.05). In SHAM and SHAM/RC, OPG was overexpressed compared to both OVX groups (both *p* < 0.05). RANKL expression was significantly lower in the SHAM/RC and OVX/RC groups compared to the SHAM group (*p* < 0.05). Thus, the RANKL/OPG ratio was significantly lower in both treated groups, SHAM RC and OVX RC, compared to the SHAM or OVX group (both *p* < 0.05). OCN expression had the highest values in the OVX/RC and SHAM groups compared to the OVX group (*p* < 0.05). Additionally, the relative gene expression of ALP was higher in the treated groups compared to the non-treated OVX group (*p* < 0.05). The molecular results are presented in Figure 3.

### 3.3. Bone Dynamics

**Figure 4:**
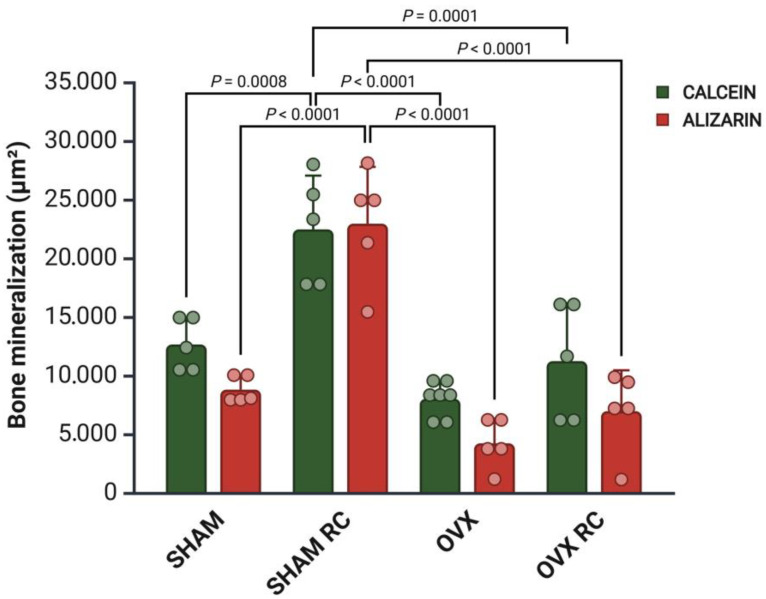
The confocal microscopy results analyzed bone dynamics: the dot–bar graph shows the mean results, and the error bars indicate the standard deviations for the fluorochromes of calcein and alizarin precipitations at 14 and 42 days after implantation, respectively. The calcein is evidence of an older bone, while the alizarin indicates a new bone at 42 days. Different values of *p* indicate statistical differences between the groups; *p* < 0.05.

The bone mineral precipitation from confocal analysis showed that RC has prophylactic and therapeutic effects in peri-implant bone dynamics. The results of fluorochrome precipitation are shown, respectively, for calcein and alizarin: SHAM/RC (23,679.18 and 22,496.17) had higher values than other groups (SHAM: 12,653.38 and 8726.04) (OVX: 8018.43 and 3763.16) (OVX/RC: 13,429.70 and 6955.64). This suggests the prophylactic effect of RC in the SHAM/RC group. In estrogen deficiency conditions, RC increased the precipitation of the old bone and new bone, improving bone remodeling in the OVX/RC group (Figure 3). A descriptive image of bone dynamics formed by overlapping the fluorochromes of calcein and alizarin in the peri-implant bone is shown in Figure 4.

**Figure 5:**
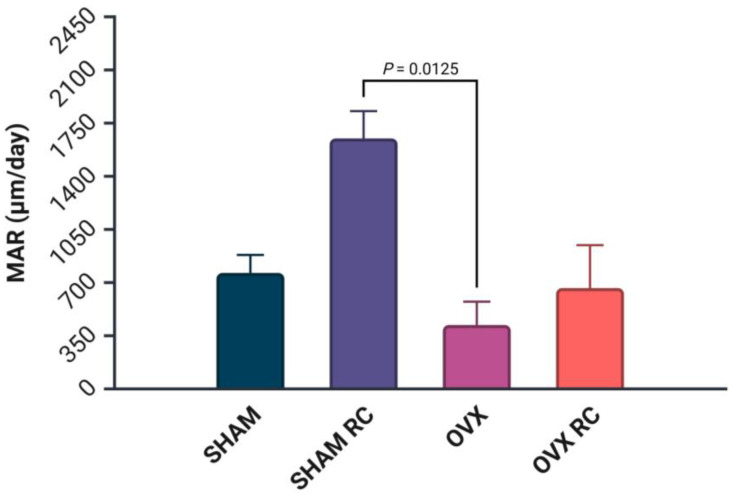
Mineral apposition rate (MAR). The MAR was measured to evaluate the rate of bone formation in response to RC treatment. Data are expressed as the distance between two fluorochrome labels (calcein and alizarin red) divided by the time interval between the injections. Results are presented as the mean ± SEM for each group. Statistical comparisons were made between groups, with a significant difference indicated by a corresponding *p* value of *p* < 0.05.

**Figure 6:**
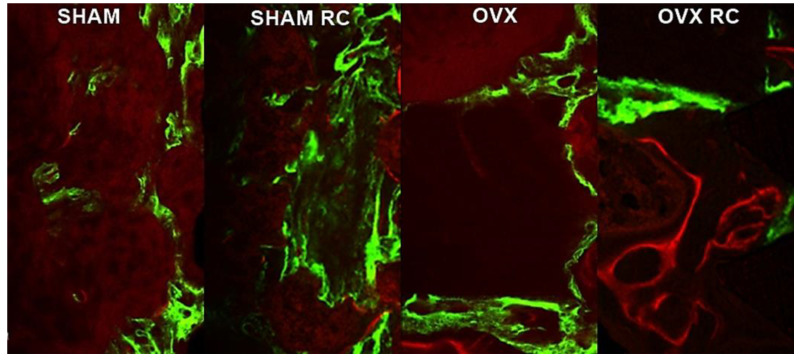
Bone dynamics. A representative image of bone dynamics formed by overlapping the fluorochromes of calcein and alizarin in the peri-implant bone of the tibia in each group for the SHAM, SHAM/RC, OVX, and OVX/RC groups after 60 days of implantation using to ImageJ software (version 1.52v, National Institutes of Health, Bethesda, MD, USA).

The MAR values are reported in micrometers per day (µm/day) in Figure 5. The results show that SHAM/RC had the highest MAR (1515 µm/day), followed by SHAM (850 µm/day), OVX/RC (698 µm/day), and OVX (400 µm/day). A significant statistical difference was observed between the SHAM/RC and OVX groups (*p* < 0.05) (see Figure 6).

### 3.4. Immunohistochemical Analysis

The Runx2 immunostaining results indicated light expression in the SHAM group, whereas moderate staining was observed in the SHAM/RC, OVX, and OVX/RC groups. This suggests that Runx2, a key transcription factor in osteoblast differentiation, may be more actively involved in both treated groups and the estrogen deficiency group.

ALP exhibited moderate staining in the SHAM, SHAM/RC, and OVX/RC groups, while the light immunolabeling in the OVX group suggests reduced bone formation under estrogen deficiency.

OPN staining was moderate in the SHAM, SHAM/RC, and OVX/RC groups, reflecting ongoing biomineralization processes, but it was light in the OVX group, further emphasizing the impact of estrogen deficiency on bone matrix protein expression.

OCN demonstrated intense staining in the SHAM/RC group, signifying robust bone formation, with moderate staining in the OVX group and light staining in both the SHAM and OVX/RC groups. This indicates that RC treatment enhances osteocalcin production, a marker of mature osteoblasts.

OPG and RANKL, proteins involved in bone remodeling, showed moderate immunolabeling in the SHAM/RC and OVX/RC groups, while light staining was noted in the SHAM and OVX groups. With the immunolabeling data, this suggests that RC not only promotes osteoblastic activity but also helps to balance bone resorption and formation.

Finally, TRAP immunolabeling, which indicates osteoclast activity, was less pronounced across all groups, suggesting that while RC promotes bone formation, it may also help mitigate excessive bone resorption in the treated groups.

**Figure 7:**
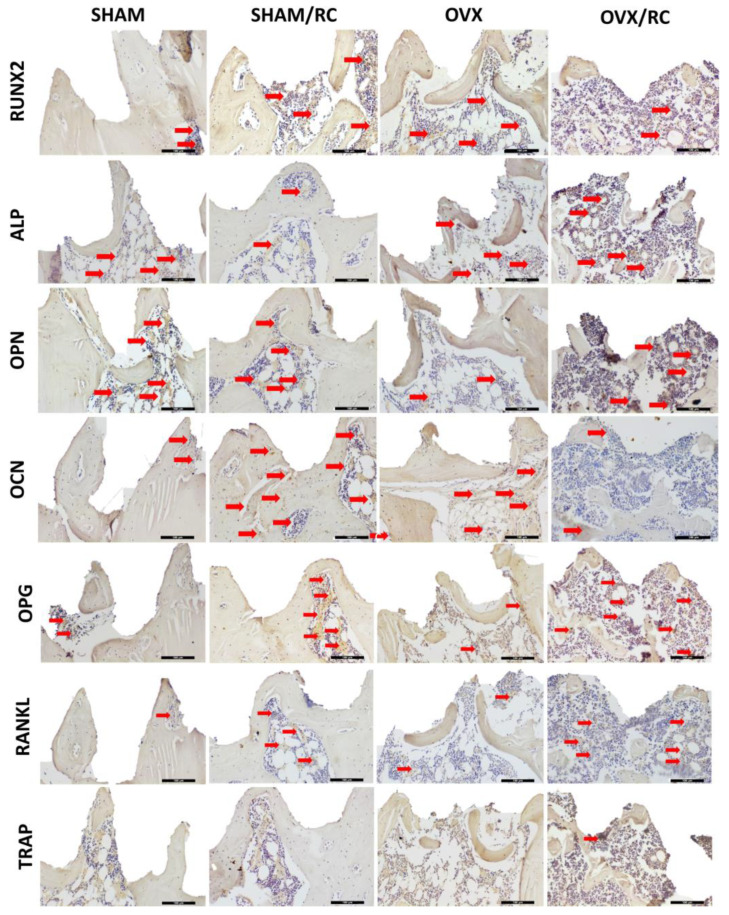
Immunolabeling in peri-implant bone healing for the SHAM, SHAM/RC, OVX, and OVX/RC groups after 60 days of implantation. The red arrows indicate positive immunostaining for the proteins Runx2, ALP, OPN, OCN, OPG, RANKL, and TRAP. Scale bar = 100 µm. Original magnification: 20×.

Overall, these findings from immunohistochemical analyses are shown in Table 2 and represented in Figure 7.

## 4. Discussion

There is an increasing demand for oral rehabilitative treatments such as dental implants due to the rising life expectancy. However, systemic conditions such as osteoporosis resulting from estrogen deficiency can impair bone healing around implants [28]. As a result, the search for natural therapeutic or preventive treatments has become more appealing. Natural compounds for treating osteoporosis may have fewer adverse effects compared to synthetic drugs [41], and patients might be more open to natural alternatives. RC has been shown to positively influence bone metabolism, promoting the differentiation of osteoblasts and the apoptosis of osteoclasts, which helps maintain the balance between bone formation and resorption [22,38]. In this sense, advances in implant dentistry have led to an increased interest in therapeutic or preventive alternatives to enhance impaired bone healing around implants [13]. Therefore, this study aimed to clarify the mechanism of action triggered by systemically administered RC in promoting bone healing around implants in healthy and ovariectomized rats.

The biomechanical analysis of removal torque was the primary outcome of this study. The results demonstrated that both the SHAM/RC and OVX/RC groups exhibited the highest removal torque values, highlighting the beneficial effect of systemic RC administration on peri-implant bone healing. Specifically, RC administration enhanced bone healing in both healthy rats and ovariectomized rats. This effect was observed under two distinct conditions: prophylactic, where there is no estrogen deficiency, and therapeutic, under estrogen deficiency. In cases of estrogen deficiency treated with RC, the removal torque values were comparable between the OVX/RC and SHAM groups. The prophylactic effect observed in the SHAM/RC group was notably superior to that in the SHAM group, suggesting that RC’s prophylactic action on bone may exceed its therapeutic effects. These effects on peri-implant bone biomechanics were further supported by cellular responses evidenced through RT-PCR analysis. Biomineralization and bone maturation were reflected in gene expression [39]. ALP expression was significantly elevated in both the SHAM/RC and OVX/RC groups compared to the OVX group, highlighting its role in phosphate precipitation within the bone matrix, which enhances the quality of hydroxyapatite crystals [42]. The OCN, another key biomineralization marker [43], showed increased gene expression in the OVX/RC group, with similar results in the SHAM and SHAM/RC groups. The balanced expression of OCN in the treated groups compared to the SHAM group indicates effective peri-implant bone healing, characterized by mature bone development with the RC treatment.

The outcomes of this study included an assessment of the immunolabeling of bone proteins. In the findings, the higher Runx2 immunolabeling in the RC groups suggests a positive effect on osteoblast differentiation, as this transcription factor is a marker of pre-osteoblasts [44]. Also, this assessment revealed moderate ALP immunostaining in the SHAM, OVX, and OVX/RC groups. In addition, the lighter ALP immunostaining in the SHAM/RC group showed that RC accelerated bone biomineralization. The moderate OPN immunostaining observed in both treated groups, OVX/RC and SHAM/RC, suggests that biomineralization in these groups resembles the physiological conditions of the SHAM group. A higher OCN in the SHAM group indicates that bone turnover was faster in prophylactic treatment compared to the other groups. Likewise, the parameters of biomineralization and bone turnover were assessed by molecular analysis using RT-PCR. In comparation, the results showed an increased expression of the OPG gene under prophylactic conditions in SHAM/RC. Importantly, OPG is involved in the OPG-RANK-RANKL resorption pathway, and its action as a RANK agonist inhibits osteoclast differentiation and indirectly affects bone formation [45]. The RANKL/OPG ratio was significantly lower in both treated groups, SHAM RC and OVX RC, compared to the untreated groups. This indicates that RC acts as an anti-catabolic agent, positively impacting bone health and peri-implant bone healing [40].

The balance of bone remodeling was evaluated by examining bone dynamics using fluorochrome labeling. The results indicated that the bone surrounding the implant showed the highest rates of new and old bone formation in both the SHAM/RC and OVX/RC groups. This fluorochrome data demonstrate the enhanced peri-implant bone dynamics facilitated by RC treatment in both healthy and estrogen-deficient conditions. It suggests that the bone formed around the implant undergoes consistent turnover, reflecting bone metabolism with concurrent bone formation and resorption during RC treatment. In the OVX/RC group, RC exhibited a therapeutic effect, significantly improving bone mineral precipitation in the bone surrounding the implant compared to the OVX group. Notably, the results in the SHAM/RC group were even more pronounced, indicating that RC could serve as an effective prophylactic treatment for enhancing bone biomineralization around implants in the presence of healthy estrogen levels.

Comparisons with previous studies are crucial for evaluating the current findings. The anabolic and anti-catabolic effects of RC have been observed in prior research [21]. In one study, RC improved the quality of trabecular bone in rats with diabetes-induced osteoporosis [21]. The anti-catabolic effect was mediated by a reduction in osteoclastic activity through RANKL [21]. This earlier research also highlighted the positive impact of RC on osteogenesis via cannabinoid receptors 1 and 2 [21]. A second study revealed its dual impact on osteoblast differentiation and osteoclast apoptosis in ovariectomized rats, also demonstrating its non-toxicity [20]. Thus, these studies demonstrate RC’s ability to improve bone metabolism and reduce osteoclastic activity in an osteoporosis model, similarly to what was observed in the OVX/RC group of the present study. In a third study, the antiresorptive effects of RC were tested on mouse bone cells [46]. This in vitro research demonstrated that RC significantly suppressed the expression of RANKL and decreased serum levels of C-terminal telopeptide fragments of type I collagen [46]. These findings, along with the current data, suggest that RC may serve as an alternative for improving peri-implant bone turnover.

Based on the findings, a deeper interpretation is warranted. In conditions of estrogen deficiency, such as those observed in the OVX/RC group, systemic treatment with RC appears to exert anti-osteoclastic effects and partially enhance osteoblastic function. This is evidenced by the significant increases in OCN and ALP but not OPN expression. The significant reduction in RANKL expression was sufficient to decrease the RANKL/OPG ratio. These changes suggest that while RC helps inhibit osteoclast activity, it may partially restore osteoblast function. In contrast, under physiological conditions where estrogen is present, as demonstrated in the SHAM/RC group, RC exhibits anabolic effects. In this scenario, RC seems to increase OPN, OCN, and ALP expression, while also reducing RANKL levels. This further decreases the RANKL/OPG ratio and correlates with the increased bone mineralization in the MAR value in SHAM/RC. This indicates the intense stimulation of bone formation in the presence of estrogen. Therefore, the results in the SHAM/RC group were even more pronounced, suggesting that RC could serve as an effective prophylactic treatment to enhance bone biomineralization around implants, particularly when estrogen levels are healthy. Despite the stronger effects observed in the presence of estrogen, RC appears to serve a dual role: inhibiting bone resorption in the context of estrogen deficiency, while simultaneously stimulating bone formation under physiological conditions. This dual action underscores the therapeutic potential of RC in managing peri-implant bone health across different hormonal environments.

Lastly, in terms of clinical relevance, it could benefit females undergoing dental implant treatment. However, it is important to note that the effects of RC in this study are currently limited to female subjects, which represents a limitation. Future studies should involve male rats to determine whether RC has sex-dependent effects on peri-implant bone healing. Also, these results should be considered with the limitations of pre-clinical studies. Although the rat tibia medullary bone is a well-established model for studying peri-implant repair, it is important to recognize that endochondral bone healing may differ from alveolar bone healing [31]. Nevertheless, the tibial model remains widely recognized for assessing osteogenesis and cellular responses relevant to osseointegration in rodent models [31,32]. Future research using mandibular models is needed to confirm our findings in the alveolar bone. We also acknowledge that while the data suggest a prophylactic effect of RC, further biomechanistic studies are needed to fully understand the underlying mechanisms, particularly the role of estrogen in modulating RC’s effect. Additionally, the pharmacokinetics of RC remain to be explored in future research to confirm its absorption, distribution, and local concentration in the bone. These aspects are essential for fully understanding its therapeutic potential.

## 5. Conclusions

In conclusion, RC enhances peri-implant bone healing and biomineralization in both healthy and ovariectomized rats. Its effects were stronger in healthy rats, suggesting that estrogen may enhance its efficacy. These findings support RC’s potential as both a prophylactic and therapeutic agent, with hormonal status influencing its effectiveness. Further research is needed to explore the mechanisms.

## Figures and Tables

**Table 1:** TaqMan probes for real-time PCR.

Gene	Gene Reference	Code Identification	Forward Primer, 5′→3′	Reverse Primer, 5′→3′
OPG	NM_057149.2	Rn00563499_m1	GCACTCCTGGTGTTCTTGGA	TTTGGTCCCAGGCAAACTGT
RANKL	NM_057149.1	Rn00589289_m1	CGAGCGCAGATGGATCCTAA	GAGCCACGAACCTTCCATCA
OCN	NM_013414.1	Rn00566386_g1	CTCTGAGTCTGACAAAGCCTTCAT	GTAGCGCCGGAGTCTATTCA
ALP	NM_013059.1	Rn00564931_m1	GAGGAACGGATCTCGGGGTA	ATGAGTTGGTAAGGCAGGGTC
ß-actin	NM_031144.3	Rn00667869_m1	CCACCATGTACCCAGGCATT	CCTAGAAGCATTTGCGGTGC

**Table 2:** The scores from the immunohistochemical analysis of peri-implant bone healing for each experimental group are as follows: light labeling—1; moderate labeling—2; and intense labeling—3.

	SHAM	SHAM/RC	OVX	OVX/RC
RUNX2	1	2	2	2
ALP	2	2	1	2
OPN	2	2	1	2
OCN	1	3	2	1
OPG	1	2	1	2
RANKL	1	2	1	2
TRAP	0	0	0	1

## Data Availability

Use Data are contained within the article.
